# Subsequent primary malignancies and acute myelogenous leukemia transformation among myelodysplastic syndrome patients treated with or without lenalidomide

**DOI:** 10.1002/cam4.721

**Published:** 2016-04-20

**Authors:** Dana E. Rollison, Kenneth H. Shain, Ji‐Hyun Lee, Shalaka S. Hampras, William Fulp, Kate Fisher, Najla H. Al Ali, Eric Padron, Jeffrey Lancet, Qiang Xu, Martha Olesnyckyj, Laurie Kenvin, Robert Knight, William Dalton, Alan List, Rami S. Komrokji

**Affiliations:** ^1^ Department of Cancer EpidemiologyMoffitt Cancer CenterTampaFlorida; ^2^ Department of Malignant HematologyMoffitt Cancer CenterTampaFlorida; ^3^ Biostatistics Shared ResourceUniversity of New Mexico Cancer CenterAlbuquerqueNew Mexico; ^4^ Department of BiostatisticsMoffitt Cancer CenterTampaFlorida; ^5^ Celgene CorporationSummitNew Jersey; ^6^ Family Personalized Medicine Institute at Moffitt Cancer Center\M2GENTampaFlorida

**Keywords:** Acute myelogenous leukemia, lenalidomide, myelodysplastic syndrome, subsequent primary malignancies

## Abstract

The few studies that have examined rates of acute myeloid leukemia (AML) transformation in lenalidomide‐treated myelodysplastic syndrome (MDS) patients have been limited to deletion 5q MDS. The association between lenalidomide and subsequent primary malignancies (SPMs) in MDS patients has not been evaluated previously. We conducted a retrospective cohort study to evaluate the risk of both SPM and AML in association with lenalidomide. A cohort of MDS patients (*n* = 1248) treated between 2004 and 2012 at Moffitt Cancer Center were identified, and incident cases of SPM and AML transformation were ascertained. Using a nested case–control design, MDS controls were 1:1 matched to SPM (*n* = 41) and AML (*n* = 150) cases, on age and date of MDS diagnosis, gender, follow‐up time, IPSS, and del (5q). Associations between lenalidomide and (1) SPM incidence and (2) AML transformation were estimated with hazards ratios (HR) and 95% confidence intervals (CIs) in the cohort and odds ratios (OR) in the case–control analysis. SPM incidence did not differ significantly between cohort MDS patients treated with (0.7 per 100 person‐years) or without lenalidomide (1.4 per 100 person‐years) (HR = 1.04, 95% CI = 0.40–2.74), whereas a significantly reduced SPM risk was observed in the case–control sample (OR = 0.03, 95% CI = <0.01–0.63). Lenalidomide was not associated with AML transformation in the cohort analysis (HR = 0.75, 95% CI = 0.44–1.27) or in the case–control analyses (OR = 1.16, 95% CI = 0.52–2.56), after adjustment for potential confounders. Lenalidomide was not associated with increased risk of SPM or AML transformation in a large cohort of MDS patients mostly including nondeletion 5q MDS.

## Introduction

Myelodysplastic syndromes (MDS) are a heterogeneous group of neoplastic, hematopoietic stem cell neoplasms 1. Clinically, they are characterized by bone marrow failure with variant cytopenias, namely macrocytic anemia and a tendency to progress to acute myeloid leukemia (AML) in 20–30% of MDS cases. The International Prognostic Scoring System (IPSS) and its revised version comprise the most widely utilized tool for risk stratification in MDS 2, 3. Patients classified as having lower risk disease are treated with the goal of alleviating symptomatic cytopenias. Conversely, for patients in the higher risk group, the goal of therapy is to alter the natural history of MDS and delay transformation to AML 4, 5. Very limited options exist for treatment of MDS, with lenalidomide and hypomethylating agents being the only modalities approved by the Food and Drug Administration (FDA) in the United States.

Lenalidomide is a second‐generation immune‐modulatory drug approved by the FDA for treatment of transfusion‐dependent, lower risk, deletion 5q (del (5q)) MDS patients with or without additional cytogenetic abnormalities 6. The MDS‐003 study established lenalidomide as a standard of care 7, with 67% of MDS patients treated with lenalidomide experiencing complete transfusion independence. For lower risk MDS patients without del (5q), the National Cancer Centers Network (NCCN) guidelines indicate lenalidomide as an option for treatment of anemia after failure of treatment with erythroid‐stimulating agents (ESA) 5, 8. The MDS‐002 study demonstrated a 26% transfusion independence rate 8.

Concern about AML progression was raised in a small cohort of European del 5q MDS patients enrolled on MDS‐003, where eight MDS patients developed AML. Progression to AML was accompanied by development of complex karyotype clonal evolution in seven of those patients 9. In a more comprehensive analysis of patients with del(5q) treated on the MDS‐001 and MDS‐003 clinical trials, cytogenetic response strongly correlated with extended survival and decreased AML progression 10. In the MDS‐004 study, AML risk was 25.1% 11. RBC‐TI for ≥8 weeks was associated with 42% reductions in the relative risk of AML progression or death (*P* = 0.048) 11. The French MDS group (GFM) reported their experience from the compassionate use of lenalidomide in lower risk MDS patients with del(5q) 12. The 4‐year cumulative incidence of AML was 9% and 15.7%, among patients treated with and without lenalidomide, respectively (*P* = 0.8) 12. No statistically significant difference was observed in the 5‐year cumulative incidence of AML (23% vs. 20%) in a retrospective analysis comparing MDS‐003 and MDS‐004 lenalidomide‐treated patients (*n* = 295) to an untreated cohort in the European registry (*n* = 125) 13. The rate of AML progression among nondel 5q MDS patients treated with lenalidomide is not known.

Development of subsequent primary malignancies (SPM) after lenalidomide is concern that was identified in three‐phase III studies of multiple myeloma (MM) among patients treated with lenalidomide in the context of melphalan exposure 14, 15, 16. A recent meta‐analysis comparing the incidence of SPMs in MM patients treated with and without lenalidomide noted a statistically significant increase in SPMs associated with lenalidomide 17. The 5‐year cumulative incidence of SPMs was 6.9% for patients receiving lenalidomide and 4.8% for those who did not receive lenalidomide, corresponding to a hazard ratio of 1.55 (95% CI = 1.03–2.34) 17. To our knowledge, no study has evaluated the risk of SPM among MDS patients treated with lenalidomide.

To investigate whether lenalidomide is associated with an increased risk of SPM and/or AML transformation in MDS patients within a clinical setting in the United States, we conducted a retrospective cohort study of MDS patients (*n* = 1248) treated with or without lenalidomide at the H. Lee Moffitt Cancer Center, Tampa, Florida in 2004–2012. In addition, we examined the association of lenalidomide with both SPM and AML transformation within a nested case–control sample of the parent cohort.

## Methods

Methods have been described in detail in the supplementary material. A brief description is given below.

### Study design and population

#### Retrospective cohort and SPM/AML verification

ICD‐O‐3 histology codes for MDS (99801, 99803, 99833, 99843, 99853, 99863, 99873, 99891, and 99893) were used to identify MDS patients, ages 18 and older, with corresponding dates of diagnoses between January 1, 2004 and December 31, 2012 in Moffitt's Cancer Registry (*n* = 1248). Demographic and clinical data were obtained from the Cancer Registry and Moffitt's electronic medical record, including IPSS, treatment administered for MDS, other cancer diagnoses, date of last contact, and vital status. Cancer diagnoses other than MDS were reviewed as potential SPM cases by two Moffitt malignant hematologists (KHS and RSK), and when there was lack of agreement as to whether the case constituted a true SPM, the two reviewers discussed the case until a consensus was reached. An SPM was defined as an MDS case that was diagnosed with subsequent cancer at least 1 day after the diagnosis of MDS and further classified as being solid tumors or hematologic malignancies. A total of 180 SPM and/or AML patients were available for analyses, including 41 SPM cases and 150 cases of AML transformation.

#### Nested case–control substudy

Controls were selected from the underlying MDS cohort and were not known to have developed an SPM/AML as of the date of SPM/AML diagnosis for the matched case. Controls were 1:1 matched to the 180 SPM/AML cases on age (< or >=60 years), gender, follow‐up time (+/– 6 months), date of diagnosis (+/– 1 year), IPSS (lower [low risk and intermediate‐1] vs. higher [intermediate‐2 and high risk]), and del (5q) presence/absence.

### Data collection

Data electronically available for the MDS cohort included demographics, WHO MDS subtype, IPSS, cytogenetic risk, previous cancers, smoking status, administered chemotherapy, bone marrow transplantation, and vital status. Additional clinical information was abstracted from medical records for the nested case–control study. The study was approved by the Institutional Review Board (IRB) and the IRB at the Florida State Department of Health. The institutional IRB granted a waiver of informed consent as there was no more than minimal risk of harm to the subjects in this retrospective observational study.

### Statistical analysis

Analyses have been described in detail in the supplementary material. Briefly, transformation to SPMs (*n* = 41) and development of AML (*n* = 150) were treated as separate outcomes. SPM and AML incidence rates and exact 95% confidence intervals (CI), based on the Poisson distribution, were calculated separately, based on person‐years of follow up from the MDS diagnosis, for the overall baseline cohort and by lenalidomide treatment group. Cox proportional hazards ratios (HRs) and 95% CIs were calculated to examine the association between lenalidomide and SPM and AML, for both the overall cohort and stratified by lower versus higher IPSS, with adjustment for age at MDS diagnosis. Lenalidomide treatment was treated as a time‐varying covariate to account for different starting dates of the treatment from the diagnosis. In the nested case–control sample, to estimate the association between lenalidomide and SPM, multivariable conditional logistic regression was used to calculate odds ratios (OR) and their 95% CIs were derived, with adjustment for variables that were observed to be statistically significantly associated with SPM and/or lenalidomide among controls.

## Results

### Cohort analysis

A total of 1248 MDS patients were followed for an average of 30.5 months with a median survival of 32.8 months. In the overall cohort, MDS patients treated with lenalidomide were slightly older than those not treated with lenalidomide (*P* = 0.06), whereas there was no difference in gender (Table 1). Four MDS patients had both an AML and SPM. Based on whether lenolidomide treatment was given prior to SPM or AML, two of these patients were included in the lenalidomide group for SPM analyses and in the ‘no lenalidomide’ group for AML analyses and vice versa for the other two patients. Lenalidomide‐treated MDS patients (*n* = 210) tended to be lower risk, with 70.0% having low‐risk or intermediate‐1 IPSS, compared to 61.7% of those not treated with lenalidomide (*P* = 0.01). There were no differences in cytogenetic risk or baseline smoking status between the lenalidomide treatment groups. The overall associations between baseline cohort characteristics and lenalidomide exposure were similar for the SPM and AML analyses (Table 1).

**Table 1:** Baseline characteristics of myelodysplastic syndrome (MDS) patients, overall and by treatment with Lenalidomide, Moffitt Cancer Center, 2004–2012

	Overall cohort *n* = 1248		SPM analysis^a^					AML transformation analysis^a^				
			Lenalidomide^a^ *n* = 210		No lenalidomide *n* = 1038			Lenalidomide^a^ *n* = 210		No lenalidomide *n* = 1038		
Characteristics	*n*	%	*n*	%	*n*	%	*P*‐value^b^	*n*	%	*n*	%	*P*‐value^b^
Age at diagnosis (years) (mean[SD])	67.1	11.6	68.9	9.2	66.7	12.0	0.06	69.0	9.2	66.7	12.0	0.05
Months of follow up (mean[SD])	30.5	24.5	38.4	25.9	28.9	23.9	<0.0001	38.2	26.0	28.9	23.9	<0.0001
Gender												
Female	447	35.9	76	36.2	371	35.8		77	36.7	370	35.7	
Male	799	64.1	134	63.8	665	64.2	0.94	133	63.3	666	64.3	0.81
IPSS at MDS diagnosis												
Missing data	58	4.6	9	4.3	49	4.7		9	4.3	49	4.7	
Available data^b^:	1190	95.4	201	95.7	989	95.3	0.86	201	95.7	989	95.3	0.86
Low risk	254	21.5	59	29.5	195	19.8		61	30.5	193	19.6	
Intermediate‐1	493	41.6	81	40.5	412	41.9		81	40.5	412	41.9	
Intermediate‐2	322	27.2	45	22.5	277	28.2		43	21.5	279	28.4	
High risk	115	9.7	15	7.5	100	10.2	0.01	15	7.5	100	10.2	0.004
Cytogenetic risk												
Good	660	55.9	115	57.2	545	55.7		117	58.2	543	55.5	
Intermediate	198	16.8	28	13.9	170	17.4		28	13.9	170	17.4	
Poor	322	27.3	58	28.9	264	27.0	0.49	56	27.9	266	27.2	0.50
Smoking status												
Never	436	38.2	73	36.3	363	38.6		73	36.3	363	38.6	
Former	609	53.3	109	54.2	500	53.1		109	54.2	500	53.1	
Current	97	8.5	19	9.5	78	8.3	0.77	19	9.5	78	8.3	0.77

Lenalidomide‐stratified cohort numbers differed slightly for the SPM and AML analyses, due to four MDS patients having differing Lenalidomide classifications for SPM or AML analysis. Two of these four patients received Lenalidomide after an SPM, but before an AML and were included in the Lenalidomide group only for the AML analysis, whereas two patients received Lenalidomide after an AML but before an SPM and were included in the Lenalidomide only for the SPM analysis.

a A total of 96 and 114 MDS patients receiving Lenalidomide as part of first course or subsequent therapy, respectively.

*P*‐values comparing the Lenalidomide versus No Lenalidomide groups based on the chi‐square test using the exact method with Monte Carlo estimation.

b Includes four MDS patients who were known to have low IPSS (1 or 2) and two MDS patients who were known to have high IPSS (2 or 3), but whose specific IPSS was not known.

A total of 41 incident SPMs were observed in the MDS cohort, 33 of which were solid tumors and 8 of which were hematological malignancies (Table 2). The SPM incidence rate in the cohort overall was 1.29 per 100 person‐years (95% CI = 0.9–1.8), with cumulative 5‐year SPM incidence rates of 5.2% and 6.1% in the lenalidomide and “no lenalidomide” group, respectively. SPM incidence was not significantly different between MDS patients treated with or without lenalidomide (log‐rank *P*‐value = 0.21) (Fig. S1), even after controlling for death as a competing risk (*P* = 0.27).

**Table 2:** Incident numbers of subsequent primary malignancies (SPM) and transformation to acute myelogenous leukemia (AML) and their associations in myelodysplastic syndrome (MDS) patients (pts) treated with or without Lenalidomide, Moffitt Cancer Center, 2004–2012

	Cohort	Incident SPM			Cohort	Incident AML transformation		
Lenalidomide treatment	*N*	*n*	%	HR (95% CI)^b^	*N* a	*n*	%	HR (95% CI)^b^
No lenalidomide	1038	36	3.5	1.00 (reference)	1038	134	12.9	1.00 (reference)
Any lenalidomide	210	5	2.4	1.04 (0.40, 2.74)	210	16	7.6	0.75 (0.44, 1.27)
SPM by type:								
Solid tumors^a^								
No lenalidomide	1038	28	2.7	1.00 (reference)				
Any lenalidomide	210	5	2.4	1.37 (0.51, 3.73)		N/A		
Hematologic malignancies^b^								
No lenalidomide	1038	8	0.8	1.00 (reference)				
Any lenalidomide	210	0	0.0	Not calculable		N/A		
MDS patients with available IPSS								
No Lenalidomide	989	33	3.3	1.00 (reference)	989	131	13.2	1.00 (reference)
Any lenalidomide	201	5	2.5	1.18 (0.44, 3.16)	201	16	8.0	0.78 (0.46, 1.33)
Any lenalidomide, adjusting for IPSS				1.16 (0.43, 3.10)^a^				0.88 (0.52, 1.50)^a^
Low risk or intermediate‐1:								
No lenalidomide	609	27	4.4	1.00 (reference)	607	64	10.5	1.00 (reference)
Any lenalidomide	140	3	2.1	0.75 (0.21, 2.59)	142	4	4.2	0.44 (0.19, 1.02)
Intermediate‐2 or high risk:								
No lenalidomide	380	6	1.6	1.00 (reference)	382	67	17.5	1.00 (reference)
Any lenalidomide	61	2	3.3	3.50 (0.70, 17.57)	59	10	16.9	1.62 (0.83, 3.16)

Hazard ratios (HR) and 95% confidence intervals (CI) adjusted for age at diagnosis in years (continuous).

Baseline cohort numbers differed slightly for the SPM and AML analysis, due to 11 MDS patients who experienced both outcomes and four MDS patients received Lenalidomide either after SPM or after AML. Based on the sequence of Lenalidomide treatment with reference to SPM or AML event,the assignment of these patients to ‘No Lenalidomide’ or ‘Lenalidomide’ group varied within the SPM and AML cohort.

a Includes seven lung, four sarcomas, three melanomas, three esophageal, three prostate, two breast, two kidney, two colon, one pancreas, one urethra, one duodenum, one thyroid, on tonsil, one gallbladder, and one Merkel cell carcinoma.

b Includes six lymphomas, one multiple myeloma, and one acute promyelocytic leukemia.

Also adjusted for higher versus lower risk IPSS; N/A = not applicable.

Of the 1248 patients in the baseline MDS cohort, 150 transformed to AML throughout the observation period corresponding to an overall AML transformation rate of 4.8 per 100 person‐years (95% CI = 4.1–5.6). By year 5 of observation, 10.3% of MDS patients treated with and 22.6% treated without lenalidomide transformed to AML, a difference that was statistically significant by the log‐rank test (*P* = 0.003) (Fig. S2). The lenalidomide‐associated reduction in AML rates was only observed among lower risk MDS patients (Fig. S3); this observation remained statistically significant after controlling for death as a competing risk (*P* = 0.002).

After adjusting for age, no association between lenalidomide treatment and risk of SPM was observed in the cohort overall (HR = 1.04, 95% CI = 0.40–2.74), or after stratification by low versus high IPSS risk groups (Table 2). Of the 33 solid tumor SPMs observed, five occurred in the lenalidomide group, compared to 28 in the “no lenalidomide” group (HR = 1.37, 95% CI = 0.51–3.73). All eight hematological SPMs occurred among patients who were not treated with lenalidomide. Lenalidomide was not associated with AML transformation overall (HR = 0.75, 95% CI = 0.44–1.27), or among patients with lower risk IPSS (HR = 0.44, 95% = 0.19–1.02) or higher risk IPSS (HR = 1.62, 95% CI = 0.83–3.16) (*P* = 0.07 for the interaction).

### Nested case–control analysis

Characteristics of the MDS‐SPM cases, MDS‐AML cases and matched controls are presented in Table S1. Mean time to SPM and AML was 17.0 and 26.9 months, respectively. MDS‐SPM cases were significantly younger than MDS controls, with no difference in months of follow up. Importantly, only two MDS‐SPM cases were del‐5q and were matched to two del‐5q controls. MDS‐SPM cases were less likely to have received azacitidine compared to controls (*P* = 0.004), whereas no differences between MDS‐SPM cases and controls were observed for other baseline characteristics (Table S1). When comparing MDS patients who experienced AML transformation to MDS‐matched controls, differences were observed for cytogenetic risk, with MDS‐AML cases having better cytogenetic risk than controls (*P* = 0.0002), but a higher percentage of bone marrow myeloblasts (*P* < 0.001) (Table S1).

Associations between clinical characteristics and lenalidomide treatment are presented for all the controls combined in Table S2. On average, MDS controls treated with lenalidomide were followed longer than those not treated with lenalidomide (*P* < 0.001). There were no significant differences between the characteristics of the lenalidomide treatment groups except for ESA use, with lenalidomide‐treated MDS patient being more likely to have received ESA compared to those not treated with lenalidomide (74% vs. 49%, *P* = 0.009).

Associations between lenalidomide treatment, SPM and AML transformation are presented in Table 3. Of the 41 MDS‐SPM cases and 41 matched MDS controls, 12.2% and 29.3% received lenalidomide, respectively, an inverse association that was statistically significant after adjustment for age, histology, ESA, and azacitidine (OR = 0.03, 95% CI = <0.01–0.63). Of the 150 MDS‐AML cases and 150 matched controls, 16.0% and 20.7%, received lenalidomide, respectively (OR = 0.70, 95% CI = 0.37–1.32). No association between lenalidomide and AML transformation was observed after adjustment for cytogenetic risk, WHO MDS subtype, percent bone marrow blasts, and ESA, overall (OR = 1.16, 95% CI = 0.52–2.56).

**Table 3:** Associations between Lenalidomide treatment, subsequent primary malignancies (SPM), and acute myeloid leukemia (AML) transformation among myelodysplastic syndrome (MDS) patients

	Cases		Controls		Unadjusted	Adjusted
Lenalidomide treatment	*n*	%	*n*	%	OR (95%CI)^a^	OR (95%CI)^b^
**MDS‐SPM (41 cases, 41 controls):**						
Overall:						
No lenalidomide	36	87.8	29	70.7	1.00 (reference)	1.00 (reference)
Any lenalidomide	5	12.2	12	29.3	0.13 (0.02–1.00)	0.03 (<0.01–0.63)
Line of therapy for lenalidomide:						
First line only	2	4.9	5	12.2	0.13 (0.01–1.75)	0.02 (<0.01–0.50)
Subsequent line	3	7.3	7	17.1	0.12 (0.01–1.40)	0.09 (<0.01–6.73)
Combination therapy:						
Lenalidomide alone	5	12.2	11	26.8	0.13 (0.02–1.00)	0.03 (<0.01–0.63)
Lenalidomide + other^c^	0	0	1	2.4	Could not be estimated	Could not be estimated
**MDS‐AML (150 cases, 150 controls):**						
Overall:						
No lenalidomide	126	84.0	119	79.3	1.00 (reference)	1.00 (reference)
Any lenalidomide	24	16.0	31	20.7	0.70 (0.37–1.32)	1.16 (0.52–2.56)
Line of therapy for lenalidomide:						
First line only	8	5.3	15	10	0.46 (0.17–1.21)	0.62 (0.19–2.03)
Subsequent line	16	10.7	16	10.7	0.92 (0.42–2.00)	1.72 (0.65–4.56)
Combination therapy:						
Lenalidomide alone	14	9.3	23	15.3	0.51 (0.23–1.13)	1.00 (0.36–2.79)
Lenalidomide + other^c^	10	6.7	8	5.3	1.12 (0.44–2.88)	1.42 (0.44–4.59)

a Odds ratio (OR) and 95% confidence interval (CI).

b MDS‐SPM estimates are adjusted for age at diagnosis in years (continuous), ESA (any vs. none), and azacitidine (any vs. none) and histology; MDS‐AML estimates are adjusted for cytogenetic risk, ESA (any vs. none), percent bone marrow myeloblasts (continuous), and histology.

c Chemotherapy, vaccine, and/or azacitidine.

AML transformation results stratified by lower versus higher IPSS are presented in Table S3. MDS‐AML cases were 81% less likely to have received any lenalidomide treatment than controls (OR = 0.33, 95% CI = 0.11–1.03), and statistically significant inverse associations with AML transformation were observed for lenalidomide given alone and as first‐line therapy only. However, these associations were attenuated after adjustment for covariates. Lenalidomide was not significantly associated with AML transformation among higher IPSS risk MDS patients.

## Discussion

Overall, lenalidomide treatment of MDS patients was not associated with an increased risk of SPMs or transformation to AML. Our findings are consistent with previous studies of AML transformation 7, 12, 18. Of note, lenalidomide‐treated MDS patients in previous studies almost exclusively harbored 5q deletions 7, 11, 12, 18, whereas the vast majority of MDS patients in this study did not. In contrast to previously reported concerns about the risk of SPM among MM patients treated with lenalidomide 14, 15, 16, 17, our findings of a null association between lenalidomide treatment and the risk of SPM/AML among MDS patients may reflect the differences in patient populations, underlying disease characteristics, and/or differing therapeutic drug combinations administered for the treatment of these two subgroups of hematological malignancies.

The current observational results are generalizable to a broader MDS patient population than previously conducted clinical trials of lenalidomide in MDS. However, the observational nature of the study design has several limitations. The cohort analysis relied on SPM and AML endpoints initially identified by the Cancer Registry, and although all events were confirmed by chart review in the case–control analysis, it is possible that SPM and AML events were underreported in the Moffitt Cancer Registry. To address the possibility that MDS patients may have been diagnosed with a SPM or an AML at an institute outside of Moffitt, and therefore may not have been recorded in Moffitt's Cancer Registry, the cohort patients were linked to the Florida Cancer Data System (FCDS), Florida's state‐wide cancer registry. Based on the FCDS data, 3.5% and 5.3% of MDS cohort patients were found to have an SPM and AML diagnosed at a non‐Moffitt facility, respectively. The proportion of missed SPMs\AMLs did not differ significantly by lenalidomide treatment, thus the misclassification was unlikely to have biased the cohort analysis. However, only Moffitt medical records were reviewed for the subset of patients selected for the nested case–control analysis, and therefore, the effects of residual misclassification on the cohort analysis cannot be assessed. Any SPM or AML events detected among MDS cases that moved outside Florida might have been missed in this study.

Confounding by indication needs to be considered, given that physicians may prescribe lenalidomide to MDS patients based on certain clinical characteristics that may also influence clinical outcomes. The nested case–control substudy was conducted to provide more information on potential confounders than was available for the full cohort. Although MDS‐SPM and MDS‐AML cases were matched to controls on IPSS (low risk or intermediate‐1 vs. intermediate‐2 or high risk), case–control differences in individual prognostic factors were still observed. For example, compared to MDS controls, MDS‐AML cases were more likely to have good cytogenetic risk, but higher percentage of bone marrow myeloblasts. Of note, lower risk MDS‐AML cases with good cytogenetics had significantly (*P* = 0.003) higher percent bone marrow myeloblasts than lower risk MDS controls with good cytogenetics, indicating that the increased percent bone marrow myeloblasts may explain the higher proportion of good cytogenetics among MDS‐AML cases compared to matched controls. Even with adjustment for cytogenetic risk, percent bone marrow myeloblasts, ESA use, and WHO MDS subtype, no significant increased risks of SPM or AML transformation were observed in association with lenalidomide. However, residual confounding by unknown factors cannot be ruled out.

In summary, we did not observe an increased risk of SPM or AML among MDS patients treated with lenalidomide. This study extends previous observations of no increased risk of AML transformation associated with lenalidomide to the broader scope of MDS patients who are treated with lenalidomide, beyond del 5q, in the clinical setting of a major cancer center. Furthermore, to the best of our knowledge, this is the first study of lenalidomide in association with SPMs among MDS patients. Results from the current study should be reassuring to MDS patients and their physicians, particularly given that lenalidomide is one of few therapies available to MDS patients. Furthermore, the lack of an increased risk in SPMs and AML transformation is a critical observation in the setting of the expanding utilization of lenalidomide in hematologic malignancies.

## Conflict of Interest

The authors would like to thank Celgene Corporation for funding the study. The Moffitt Cancer Center has entered into a license agreement with Celgene Corporation. Payments have been distributed to the Moffitt Cancer Center and Moffitt Cancer Center inventors of the licensed technology which is not a part of this study. The Moffitt Cancer Center and those inventors are also entitled to receive additional payments related to the licensed technology. The Principal Investigator DER is not an inventor of the licensed technology.

The following authors have conflict of interest:

Dana Rollison, Ji‐Hyun Lee, Shalaka Hampras, William Fulp and Kate Fisher: grant from Celgene, during the conduct of the study. Kenneth Shain: Grants from Celgene, during the conduct of the study; grants and personal fees from Amgen/Onyx, personal fees from Takeda Pharmaceuticals, personal fees from Celgene, personal fees and other from Jansen, outside the submitted work. Lancet Jeffrey: Consultant, Celgene and clinical trial funding, Celgene. Robert Knight, Marta Olesnyckyj, Qiang Xu and Laurie Kenvin: Employee of Celgene and stock options in a publicly traded company. William Dalton: Unpaid board member of the following boards: Institute of Human and Machine Cognition, Personalized Medicine Coalition, National Leukemia and Lymphoma Society and, M2Gen, a for‐profit board in personalized medicine. Alan list: Consultant for Celgene. Rami Komrokji: Grants and personal fees from Celgene.

## Supporting information


**Figure S1.** Incidence of subsequent primary malignancies (SPM) among myelodysplastic syndrome (MDS) patients treated with or without Lenalidomide, Moffitt Cancer Center, 2004–2012.

**Figure S2**. Incidence of acute myelogenous leukemia (AML) among myelodysplastic syndrome (MDS) patients treated with or without Lenalidomide, at Moffitt Cancer Center, 2004–2012.

**Figure S3.** Incidence of acute myelogenous leukemia (AML) among myelodysplastic syndrome (MDS) patients treated with or without Lenalidomide and stratified by lower risk IPSS (low risk or intermediate‐1) versus higher risk IPSS (intermediate‐2 or high risk), Moffitt Cancer Center, 2004–2012.

**Table S1.** Characteristics of myelodysplastic syndrome (MDS)‐subsequent primary malignancy (SPM) cases, MDS‐AML transformation cases, and MDS‐matched controls.

**Table S2.** Association of clinical and demographic variables with Lenalidomide (Len) use among MDS controls (for SPM and AML) at Moffitt Cancer Center, 2004–2012.

**Table S3.** Associations between Lenalidomide treatment and AML transformation among MDS patients by IPSS.
