# Supplementary figures and images for: Subsequent primary malignancies and acute myelogenous leukemia transformation among myelodysplastic syndrome patients treated with or without lenalidomide
Source: Cancer Med. 2016 Apr 20;5(7):1694–701. doi: 10.1002/cam4.721 (PMC4944897; doi:10.1002/cam4.721)

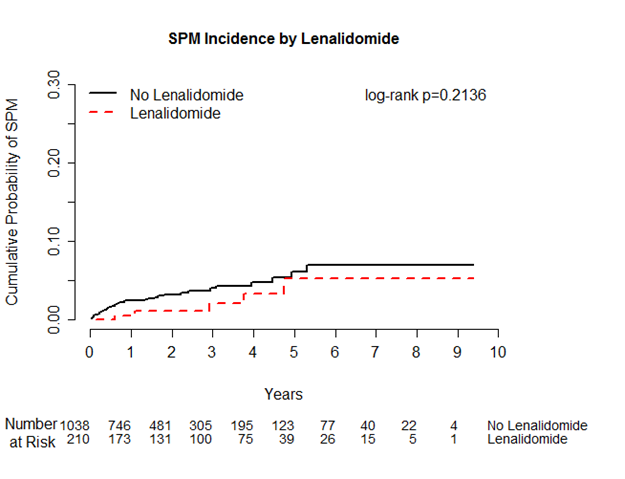

Supplement: Supplementary file 1 — Figure S1. Incidence of subsequent primary malignancies (SPM) among myelodysplastic syndrome (MDS) patients treated with or without Lenalidomide, Moffitt Cancer Center, 2004–2012. [file CAM4-5-1694-s001.tif]

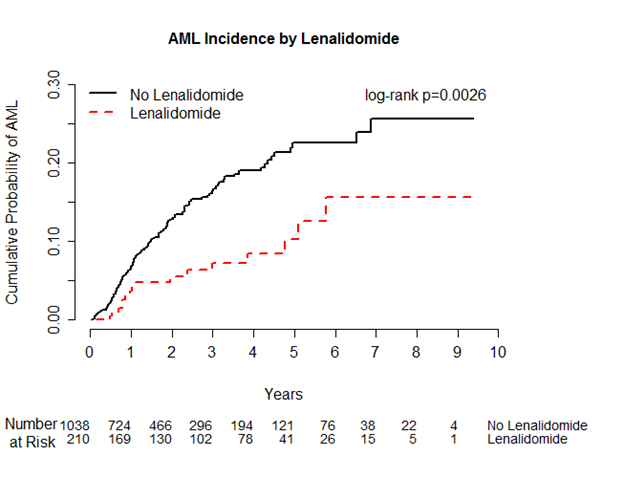

Supplement: Supplementary file 2 — Figure S2. Incidence of acute myelogenous leukemia (AML) among myelodysplastic syndrome (MDS) patients treated with or without Lenalidomide, at Moffitt Cancer Center, 2004–2012. [file CAM4-5-1694-s002.tif]

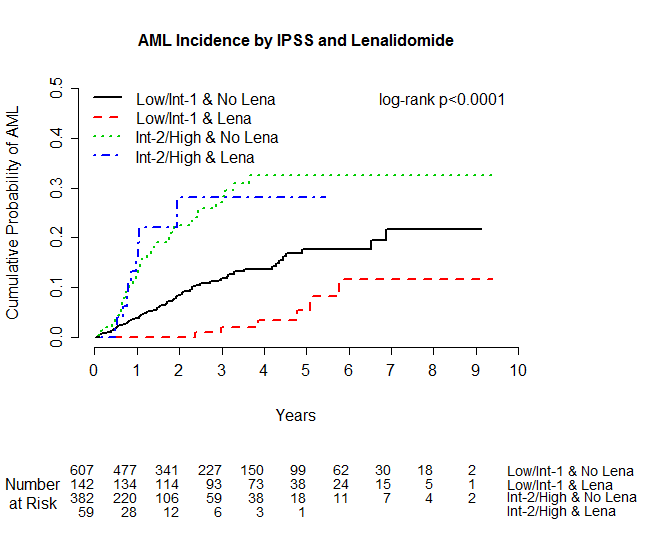

Supplement: Supplementary file 3 — Figure S3. Incidence of acute myelogenous leukemia (AML) among myelodysplastic syndrome (MDS) patients treated with or without Lenalidomide and stratified by lower risk IPSS (low risk or intermediate‐1) versus higher risk IPSS (intermediate‐2 or high risk), Moffitt Cancer Center, 2004–2012. [file CAM4-5-1694-s003.tif]
