# Supplementary material for: Subsequent primary malignancies and acute myelogenous leukemia transformation among myelodysplastic syndrome patients treated with or without lenalidomide
Source: Cancer Med. 2016 Apr 20;5(7):1694–701. doi: 10.1002/cam4.721 (PMC4944897; doi:10.1002/cam4.721)
Supplement: Supplementary file 4 — Table S1. Characteristics of myelodysplastic syndrome (MDS)‐subsequent primary malignancy (SPM) cases, MDS‐AML transformation cases, and MDS‐matched controls. [file CAM4-5-1694-s004.docx]

**Supplementary Table I. Characteristics of myelodysplastic syndrome (MDS)-subsequent primary malignancy (SPM) cases, MDS-AML transformation cases and MDS matched controls**

|  | MDS-SPM cases (n=41) | |  | MDS controls (n=41) | |  | MDS-AML cases  (n=150) | |  | MDS controls (n=150) | |  |
| --- | --- | --- | --- | --- | --- | --- | --- | --- | --- | --- | --- | --- |
| Characteristic | n | % |  | n | % | p-value^1^ | n | % |  | n | % | p-value^1^ |
| Age at MDS diagnosis in years^2^ (mean, SD) | 68.5 | 10.9 |  | 71.6 | 10.3 | 0.03 | 68.3 | 9.2 |  | 66.9 | 11.1 | 0.10 |
|  |  |  |  |  |  |  |  |  |  |  |  |  |
| Duration of Lenalidomide use (mean,SD) | 7.9 | 14.5 |  | 14.5 | 16.8 | 0.26 | 7.7 | 11.8 |  | 7.6 | 13.2 | 0.53 |
|  |  |  |  |  |  |  |  |  |  |  |  |  |
| Follow-up time in months (mean, SD) | 38.6 | 25.0 |  | 39.1 | 25.5 | 0.36 | 28.4 | 19.4 |  | 28.6 | 20.1 | 0.57 |
|  |  |  |  |  |  |  |  |  |  |  |  |  |
| Time to SPM\AML (mean,[SD]) | 17.0 | 18.7 |  |  |  |  | 26.9 | 19.2 |  |  |  |  |
| Gender^2^ |  |  |  |  |  |  |  |  |  |  |  |  |
| female | 15 | 36.6 |  | 14 | 34.1 |  | 46 | 30.7 |  | 53 | 35.3 |  |
| male | 26 | 63.4 |  | 27 | 65.9 | 1.00 | 104 | 69.3 |  | 97 | 64.7 | 0.14 |
|  |  |  |  |  |  |  |  |  |  |  |  |  |
| IPSS at MDS diagnosis^2^ |  |  |  |  |  |  |  |  |  |  |  |  |
| Low risk or intermediate-1 | 30 | 73.2 |  | 31 | 75.6 |  | 70 | 46.7 |  | 72 | 48.0 |  |
| Intermediate-2 or high risk | 8 | 19.5 |  | 9 | 22.0 |  | 77 | 51.3 |  | 78 | 52.0 |  |
| Missing | 3 | 7.3 |  | 1 | 2.4 | 1.00 | 3 | 2.0 |  | 0 | 0 | 0.50 |
|  |  |  |  |  |  |  |  |  |  |  |  |  |
| Cytogenetic risk |  |  |  |  |  |  |  |  |  |  |  |  |
| Good | 29 | 70.7 |  | 23 | 56.1 |  | 81 | 54.0 |  | 52 | 34.7 |  |
| Intermediate | 3 | 7.3 |  | 10 | 24.4 |  | 25 | 16.7 |  | 37 | 24.7 |  |
| Poor | 4 | 9.8 |  | 7 | 17.1 |  | 41 | 27.3 |  | 59 | 39.3 |  |
| Missing | 5 | 12.2 |  | 1 | 2.4 | 0.12 | 3 | 2.0 |  | 2 | 1.3 | 0.0002 |
|  |  |  |  |  |  |  |  |  |  |  |  |  |
| Histology^3^ |  |  |  |  |  |  |  |  |  |  |  |  |
| Refractory anemia (RA) | 7 | 17.1 |  | 9 | 22.0 |  | 6 | 4.0 |  | 11 | 7.3 |  |
| Refractory anemia with sideroblasts | 6 | 14.6 |  | 1 | 2.4 |  | 5 | 3.3 |  | 15 | 10.0 |  |
| Refractory anemia with excess blasts | 11 | 26.8 |  | 16 | 39.0 |  | 104 | 69.3 |  | 64 | 42.7 |  |
| Refractory cytopenia with multilineage dysplasia | 7 | 17.1 |  | 13 | 31.7 |  | 24  24 | 16.0  16.0 |  | 54  54 | 36.0  36.0 |  |
| MDS with 5q deletion | 2 | 4.9 |  | 2 | 4.9 |  | 2 | 1.3 |  | 2 | 1.3 |  |
| MDS, not otherwise specified | 8 | 19.5 |  | 0 | 0 | 0.008 | 9 | 6.0 |  | 4 | 2.7 | <0.0001 |

**Supplementary Table I. (continued). Characteristics of myelodysplastic syndrome (MDS)-subsequent primary malignancy (SPM) cases, MDS-AML transformation cases and MDS matched controls**

|  | MDS-SPM cases (n=41) | |  | MDS controls (n=41) | |  | MDS-AML cases  (n=150) | |  | MDS controls (n=150) | |  |
| --- | --- | --- | --- | --- | --- | --- | --- | --- | --- | --- | --- | --- |
| Characteristic | n | % |  | n | % | p-value^1^ | n | % |  | n | % | p-value^1^ |
| % peripheral blood myeloblasts |  |  |  |  |  |  |  |  |  |  |  |  |
| None | 34 | 82.9 |  | 39 | 95.1 |  | 118 | 78.7 |  | 133 | 88.7 |  |
| Any | 2 | 4.9 |  | 2 | 4.9 |  | 29 | 19.3 |  | 17 | 11.3 |  |
| Missing | 5 | 12.2 |  | 0 | 0 | 1.00 | 3 | 2.0 |  | 0 | 0 | 0.07 |
| % bone marrow myeloblasts (mean, [SD]) | 4.6 | 4.4 |  | 3.2 | 3.0 | 0.13 | 7.9 | [5.0] |  | 5.5 | 4.9 | <0.001 |
|  |  |  |  |  |  |  |  |  |  |  |  |  |
| Erythroid stimulating agents |  |  |  |  |  |  |  |  |  |  |  |  |
| No | 13 | 31.7 |  | 10 | 24.4 |  | 73 | 48.7 |  | 74 | 49.3 |  |
| Yes | 28 | 68.3 |  | 31 | 75.6 | 0.51 | 77 | 51.3 |  | 76 | 50.7 | 1.00 |
|  |  |  |  |  |  |  |  |  |  |  |  |  |
| Azacitidine |  |  |  |  |  |  |  |  |  |  |  |  |
| None | 31 | 75.6 |  | 18 | 43.9 |  | 49 | 32.7 |  | 52 | 34.7 |  |
| Any | 10 | 24.4 |  | 23 | 56.1 | 0.004 | 101 | 67.3 |  | 98 | 65.3 | 0.78 |
|  |  |  |  |  |  |  |  |  |  |  |  |  |
| Smoking status |  |  |  |  |  |  |  |  |  |  |  |  |
| Never | 14 | 34.1 |  | 17 | 41.5 |  | 62 | 41.3 |  | 58 | 38.7 |  |
| Former | 22 | 53.7 |  | 21 | 51.2 |  | 76 | 50.7 |  | 74 | 49.3 |  |
| Current | 5 | 12.2 |  | 3 | 7.3 |  | 11 | 7.3 |  | 17 | 11.3 |  |
| Missing | 0 | 0 |  | 0 | 0 | 0.72 | 1 | 0.7 |  | 1 | 0.7 | 0.44 |
|  |  |  |  |  |  |  |  |  |  |  |  |  |
| History of cancer |  |  |  |  |  |  |  |  |  |  |  |  |
| No | 31 | 75.6 |  | 29 | 70.7 |  | 109 | 72.7 |  | 100 | 66.7 |  |
| Yes | 10 | 24.4 |  | 12 | 29.3 | 0.80 | 41 | 27.3 |  | 50 | 33.3 | 0.31 |
|  |  |  |  |  |  |  |  |  |  |  |  |  |
| Family history of cancer |  |  |  |  |  |  |  |  |  |  |  |  |
| No | 15 | 36.6 |  | 22 | 53.7 |  | 64 | 42.7 |  | 72 | 48.0 |  |
| Yes | 26 | 63.4 |  | 19 | 46.3 | 0.17 | 86 | 57.3 |  | 78 | 52.0 | 0.40 |

^1^ Univariable conditional logistic regression with exact Score test for all variables except ‘Duration of Lenalidomide use’ for which Wilcoxon’s test was used; ^2^ Of the 180 case-control pairs matched, one or more matching criteria had to be relaxed for 25 pairs, including 12 pairs with different age category, 18 pairs of different gender, 2 pairs with dates of MDS diagnosis greater than 365 days apart, and 5 pairs in which one MDS patient had missing IPSS and the other did not; ^3^p-values were calculated based on three categories of histology: 1) refractory anemia, refractory anemia with ring sideroblasts, MDS del-5q, and MDS not otherwise specified, 2) refractory cytopenia with multilineage dysplasia, and 3) refractory anemia with excess blasts
