# Supplementary material for: Subsequent primary malignancies and acute myelogenous leukemia transformation among myelodysplastic syndrome patients treated with or without lenalidomide
Source: Cancer Med. 2016 Apr 20;5(7):1694–701. doi: 10.1002/cam4.721 (PMC4944897; doi:10.1002/cam4.721)
Supplement: Supplementary file 5 — Table S2. Association of clinical and demographic variables with Lenalidomide (Len) use among MDS controls (for SPM and AML) at Moffitt Cancer Center, 2004–2012. [file CAM4-5-1694-s005.docx]

**Supplementary Table II. Association of clinical and demographic variables with Lenalidomide (Len) use among MDS controls (for SPM and AML) at Moffitt Cancer Center, 2004-2012**

|  | | Any Len (n=42) | |  | No Len (n=138) | | p-value^1^ |
| --- | --- | --- | --- | --- | --- | --- | --- |
| Characteristics | | n | % |  | n | % |  |
| Age at Diagnosis (years) (mean,SD) | | 70.3 | 9.2 |  | 67.2 | 11.8 | 0.10 |
|  |  |  |  |  |  |  |  |
| Months of follow up (mean, SD) | | 40.7 | 23.2 |  | 27.9 | 20.3 | <0.001 |
|  |  |  |  |  |  |  |  |
| IPSS^2^ | |  |  |  |  |  |  |
|  | Intermediate-2 or high risk | 15 | 35.7 |  | 67 | 48.6 | 0.170 |
|  | Low risk or intermediate-1 | 27 | 64.3 |  | 70 | 50.7 |  |
|  | |  |  |  |  |  |  |
| Histology^3^ | |  |  |  |  |  |  |
|  | Refractory anemia (RA) | 3 | 7.1 |  | 16 | 11.6 | 0.056 |
|  | RA with sideroblasts (RARS) | 10 | 23.8 |  | 6 | 4.3 |  |
|  | RA with excess blasts | 14 | 33.3 |  | 61 | 44.2 |  |
|  | Refractory cytopenia w/multilineage dysplasia | 12 | 28.6 |  | 50 | 36.2 |  |
|  | MDS with 5q deletion | 3 | 7.1 |  | 1 | 0.7 |  |
|  | MDS, not otherwise specified (MDS-NOS) | 0 | 0 |  | 4 | 2.9 |  |
|  |  |  |  |  |  |  |  |
| Gender | |  |  |  |  |  |  |
|  | Female | 20 | 47.6 |  | 44 | 31.9 | 0.077 |
|  | Male | 22 | 52.4 |  | 94 | 68.1 |  |
|  |  |  |  |  |  |  |  |
| Smoking status^4^ | |  |  |  |  |  |  |
|  | Never | 22 | 52.4 |  | 48 | 34.8 | 0.108 |
|  | Former | 15 | 35.7 |  | 74 | 53.6 |  |
|  | Current | 5 | 11.9 |  | 15 | 10.9 |  |
|  |  |  |  |  |  |  |  |
| History of cancer | |  |  |  |  |  |  |
|  | No | 29 | 69.0 |  | 93 | 67.4 | 0.867 |
|  | Yes | 13 | 31.0 |  | 45 | 32.6 |  |
|  | |  |  |  |  |  |  |
| Family history of cancer | |  |  |  |  |  |  |
|  | No | 19 | 45.2 |  | 66 | 47.8 | 0.871 |
|  | Yes | 23 | 54.8 |  | 72 | 52.2 |  |
|  | |  |  |  |  |  |  |
| Cytogenetic risk^5^ | |  |  |  |  |  |  |
|  | Good | 22 | 52.4 |  | 50 | 36.2 | 0.149 |
|  | Intermediate | 9 | 21.4 |  | 34 | 24.6 |  |
|  | Poor | 10 | 23.8 |  | 52 | 37.7 |  |
|  | |  |  |  |  |  |  |
| Peripheral blood myeloblasts (%) | |  |  |  |  |  |  |
|  | No | 36 | 85.7 |  | 125 | 90.6 | 0.405 |
|  | Yes | 6 | 14.3 |  | 13 | 9.4 |  |
|  |  |  |  |  |  |  |  |
| Erythroid stimulating agents | |  |  |  |  |  |  |
|  | No | 11 | 26.2 |  | 70 | 50.7 | 0.009 |
|  | Yes | 31 | 73.8 |  | 68 | 49.3 |  |
|  | |  |  |  |  |  |  |
| Azacitidine | |  |  |  |  |  |  |
|  | No Azacitidine | 15 | 35.7 |  | 50 | 36.2 | 1.0 |
|  | Azacitidine | 27 | 64.3 |  | 88 | 63.8 |  |
|  | |  |  |  |  |  |  |
| % Bone marrow myeloblasts (mean [SD]) | | 4.3 | [4.0] |  | 5.4 | [4.9] | 0.357 |

^1^Simulated Pearson Chi-Square test for categorical or Wilcoxon sum rank test for continuous variables; ^2^IPSS data missing for 1 patient in the No Len group; ^3^p-values were calculated based on three categories of histology: 1) refractory anemia, refractory anemia with ring sideroblasts, MDS del-5q, and MDS not otherwise specified, 2) refractory cytopenia with multilineage dysplasia, and 3) refractory anemia with excess blasts;  ^4^smoking status missing for 1 patient in the No Len group; ^5^cytogenetic risk missing for 1 patient in the Any Len and 2 patients in the No Len groups, respectively
