# Supplementary material for: Subsequent primary malignancies and acute myelogenous leukemia transformation among myelodysplastic syndrome patients treated with or without lenalidomide
Source: Cancer Med. 2016 Apr 20;5(7):1694–701. doi: 10.1002/cam4.721 (PMC4944897; doi:10.1002/cam4.721)
Supplement: Supplementary file 6 — Table S3. Associations between Lenalidomide treatment and AML transformation among MDS patients by IPSS. [file CAM4-5-1694-s006.docx]

**Supplementary Table III. Associations between Lenalidomide treatment and AML transformation among MDS patients by IPSS**

|  | MDS-AML cases  n (%) | MDS controls  n (%) | Unadjusted OR (95%CI) | Adjusted OR (95% CI)^1^ |
| --- | --- | --- | --- | --- |
| Low risk or intermediate-1 IPSS: | n=70 | n=70 |  |  |
| Overall: |  |  |  |  |
| No Lenalidomide | 60(85.7%) | 52(74.3%) | 1.00 (reference) | 1.00 (reference) |
| Any Lenalidomide | 10(14.3%) | 18(25.7%) | 0.33 (0.11-1.03) | 0.44 (0.10-1.94) |
|  |  |  |  |  |
| Line of Lenalidomide therapy: |  |  |  |  |
| First line only | 4(5.7%) | 12(17.1%) | 0.19 (0.04-0.88) | 0.26 (0.04-1.92) |
| Subsequent line | 6(8.6%) | 6(8.6%) | 0.68 (0.15-3.04) | 0.68 (0.11-4.22) |
|  |  |  |  |  |
| Combination therapy: |  |  |  |  |
| Lenalidomide alone | 18(25.7%) | 7(10%) | 0.17 (0.04-0.74) | 0.19 (0.03-1.25) |
| Lenalidomide + other | 0(0%) | 3(4.3%) | Could not be estimated | Could not be estimated |
|  |  |  |  |  |
| Intermediate-2 or high risk IPSS: | n=77 | n=77 |  |  |
| Overall: |  |  |  |  |
| No Lenalidomide | 63(81.8%) | 64(83.1%) | 1.00 (reference) | 1.00 (reference) |
| Any Lenalidomide | 14(18.2%) | 13(16.9%) | 1.09 (0.48-2.47) | 2.06 (0.69-6.18) |
|  |  |  |  |  |
| Line of Lenalidomide therapy: |  |  |  |  |
| First line only | 4(5.2%) | 3(3.9%) | 1.34 (0.30-6.01) | 1.84 (0.28-12.00) |
| Subsequent line | 10(13%) | 10(13%) | 1.02 (0.40-2.57) | 2.15 (0.62-7.41) |
|  |  |  |  |  |
| Combination therapy: |  |  |  |  |
| Lenalidomide alone | 7(9.1%) | 5(6.5%) | 1.38 (0.43-4.40) | 3.69 (0.81-16.73) |
| Lenalidomide + other | 7(9.1%) | 8(10.4%) | 0.91 (0.33-2.55) | 1.21 (0.30-4.87) |

^1^ adjusted for cytogenetic risk, ESA use, percent bone marrow myeloblasts (continuous), and histology
